# Pregnancy outcome in women with Gaucher disease type 1 who had unplanned pregnancies during eliglustat clinical trials

**DOI:** 10.1002/jmd2.12172

**Published:** 2020-10-18

**Authors:** Elena Lukina, Manisha Balwani, Nadia Belmatoug, Nora Watman, Derralynn Hughes, Sebastiaan J. M. Gaemers, Meredith C. Foster, Grace Lewis, M. Judith Peterschmitt

**Affiliations:** ^1^ National Research Center for Hematology Moscow Russia; ^2^ Icahn School of Medicine at Mount Sinai New York New York USA; ^3^ Beaujon Hospital, University of Paris, Assistance Publique‐Hopitaux de Paris Paris France; ^4^ Hospital Ramos Mejia Buenos Aires Argentina; ^5^ Royal Free London NHS Foundation Trust, University College London London UK; ^6^ Sanofi Genzyme Amsterdam The Netherlands; ^7^ Sanofi Genzyme Cambridge Massachusetts USA

**Keywords:** clinical trials, eliglustat, Gaucher disease, lysosomal storage disorder, pregnancy

## Abstract

Gaucher disease type 1 (GD1) is an inherited lysosomal storage disorder caused by deficient enzymatic activity of acid β‐glucosidase, resulting in accumulation of its substrate glucosylceramide, leading to debilitating visceral, hematologic, and skeletal manifestations. Women with GD1 are at increased risk for complications during pregnancy, delivery, and postpartum. Treatment with enzyme replacement therapy is generally recommended before and during pregnancy to reduce risks. Eliglustat, an oral substrate‐reduction therapy, is a first‐line treatment for adults with GD1 adults who have extensive, intermediate, or poor CYP2D6‐metabolizer phenotypes (>90% of patients). We report on pregnancy outcomes among women in eliglustat trials who had unplanned pregnancies and female partners of men in the trials. In four phase 2 and 3 eliglustat trials of 393 adults with GD1, women of childbearing potential were required to use contraception, have monthly pregnancy tests, and discontinue eliglustat promptly if pregnant. In phase 2 and 3 trials, 18 women had 19 pregnancies, resulting in 14 healthy infants from 13 pregnancies (one set of twins), three elective terminations, one ectopic pregnancy, one spontaneous abortion, and one in utero death. Median estimated eliglustat exposure duration during pregnancy was 38 days. In phase 1 trials (non‐GD1 subjects), one woman had a spontaneous abortion. Partners of 16 eliglustat‐treated men with GD1 had 18 pregnancies, all resulting in healthy infants. Eliglustat is not approved during pregnancy due to limited data. Guidelines for clinicians and patients with GD that address use of eliglustat in women of childbearing potential are needed.

**Synopsis**

We report on outcomes of unplanned pregnancies among women treated with eliglustat for Gaucher disease type 1 who participated in phase 2 and 3 trials.

## 1 INTRODUCTION

Gaucher disease type 1 (GD1) is an autosomal recessive lysosomal storage disorder characterized by deficient activity of the enzyme acid β‐glucosidase. The resulting progressive substrate accumulation in lysosomes leads to debilitating visceral, hematologic, and skeletal manifestations.^1^ Two first‐line treatment approaches are available for GD1. Intravenous enzyme replacement therapy (ERT), first with acid β‐glucosidase purified from human placenta and later with recombinant human acid β‐glucosidase, has been available since 1991. Oral substrate‐reduction therapy (SRT) with eliglustat (Cerdelga, Sanofi Genzyme, Cambridge, Massachusetts) was approved in the United States in 2014 and in the European Union (EU) in 2015 as a first‐line treatment for adults with GD1 who have poor, intermediate, or extensive CYP2D6 metabolizer phenotypes (>90% of patients^2^).^3^, ^4^

Women with GD1 are at increased risk of complications during pregnancy, delivery, and the postpartum period.^5^ Anemia and thrombocytopenia may worsen above that normally associated with pregnancy, and excessive bleeding may complicate pregnancy, delivery, and postpartum.^5^, ^6^, ^7^ Hepatosplenomegaly may interfere with normal fetal growth.^5^, ^6^ Increased calcium demand during pregnancy further increases the risk of bone crises, osteopenia, osteonecrosis, and fractures.^5^ In clinical practice, ERT is generally recommended before and during pregnancy to mitigate pregnancy‐related risks, especially bleeding during delivery and postpartum.^5^, ^8^, ^9^ Multiple studies and reviews have been published on the use of ERT during pregnancy.^5^, ^6^, ^7^, ^10^, ^11^ Conversely, eliglustat is not approved for use in pregnancy due to lack of adequate or well‐controlled studies in pregnant women.^3^ The US Prescribing Information (PI) states that available data are not sufficient to assess drug‐associated risks of major birth defects, miscarriage, or adverse maternal or fetal outcomes,^3^ and the EU SmPC states that, as a precautionary measure, it is recommended to avoid the use of eliglustat during pregnancy.^4^

Eliglustat partially inhibits the enzyme glucosylceramide synthase, thus decreasing production of the substrate glucosylceramide and allowing the patients' residual acid β‐glucosidase activity to reduce accumulated glucosylceramide and thereby ameliorate symptoms.^12^ Eliglustat is metabolized mainly by CYP2D6 and to a lesser extent CYP3A4. Median time to maximum plasma concentration is 1.5 to 3 hours after administration.^3^ The elimination half‐life is 4 to 7 hours in nonpoor metabolizers and 9 hours in poor metabolizers.^4^ Preclinical animal data suggest no direct or indirect reproductive toxicity at the recommended human dose. In rats, eliglustat doses 6 times the recommended human dose caused a spectrum of various developmental anomalies; the eliglustat‐related effects on fetal rats were observed in association with signs of maternal toxicity.^3^ In pregnant rabbits, doses 10‐fold higher than the recommended human dose did not cause fetal harm.^3^ Placental transfer of eliglustat and its metabolites was shown in the rat; at 2 and 24 hours postdose, 0.034% and 0.013% of labeled dose was detected in fetal tissue, respectively.^4^ It is not known whether eliglustat is present in human milk, although in rats, 0.23% of labeled dose was transferred to pups during 24 hours postdose.^4^ In mature male rats, systemically nontoxic doses had no effect on sperm parameters.^4^

The eliglustat clinical trial program is the largest clinical development program in GD. Four clinical trials of eliglustat (one phase 2 and three phase 3), involving a total of 393 adults with GD1 from 29 countries, were completed. Together, these trials showed long‐term clinical benefit in treatment‐naïve patients and long‐term clinical stability in patients switching from ERT to eliglustat.^13^, ^14^, ^15^, ^16^, ^17^, ^18^, ^19^, ^20^ There was a total of 1400 patient‐years of exposure in the phase 2 and phase 3 trials with a mean treatment duration of 3.6 years on eliglustat.^21^ In addition, more than 450 participants, none with GD, were exposed to eliglustat in phase 1 trials. Most phase 1 studies were of short duration. In all trials, female participants of childbearing potential were required to use contraception. Of note, a drug‐drug interaction study found that eliglustat does not interact with oral contraceptives. Based on that study, eliglustat and oral contraceptives can be safely administered together without concern for clinically relevant drug‐drug interaction.^22^

Women of childbearing potential represent a large segment of the GD1 population that may be eligible for eliglustat. Additionally, there is interest within the GD community to learn more about potential risks of short‐term exposure to eliglustat during pregnancy. We report outcomes of unplanned pregnancies occurring in the eliglustat clinical trials.

## 2 METHODS

### 2.1 Data source

This analysis used the populations from phase 1, 2, and 3 eliglustat clinical trials (NCT00358150 [phase 2], NCT00891202 [ENGAGE], NCT00943111 [ENCORE], NCT01074944 [EDGE]) to evaluate pregnancy outcomes during the trials. All patients in these trials had a confirmed diagnosis of GD1 based on enzymatic testing and absence of neurologic symptoms compatible with a diagnosis of Gaucher disease type 3. The protocols for all studies were approved by the institutional review boards or independent ethics committees of participating institutions. Women of childbearing potential were required to use contraception throughout their respective study and perform pregnancy tests at least monthly. Either a barrier method or hormonal contraceptive with ethinyl estradiol and norethindrone or similar active components were considered acceptable forms of contraception. In the case of a confirmed pregnancy, the female participant stopped eliglustat and was discontinued from the study. As per the trial protocols, progress of all pregnancies, either in female patients or female partners of male patients, was followed until the outcome of the pregnancy was known. Male participants were required to use contraception during the phase 1 drug‐drug interaction study of eliglustat and acid‐reducing agents. Initially, male participants were required to use contraception during the phase 2, ENGAGE, and ENCORE trials, but the requirement was later removed with protocol amendments. Male participants were not required to use contraception at any time during the EDGE trial. Information about infants beyond pregnancy outcome, such as long‐term effects of exposure during pregnancy, is not available.

### 2.2 Analysis

This is a descriptive analysis of pregnancy outcomes in eliglustat clinical trials with respect to three groups: women with GD1 in phase 2 or phase 3 trials, female partners of men with GD1 in phase 2 or 3 trials, and a woman without GD1 in a phase 1 trial.

For women with GD1 who became pregnant while taking eliglustat during phase 2 and phase 3 clinical trials, in addition to pregnancy outcome, we retrospectively analyzed data collected during the clinical trials, such as age at diagnosis of GD, age at first eliglustat exposure, total time on eliglustat, GD genotype, CYP2D6 metabolizer phenotype, race, splenectomy status, prior treatment status (treatment‐naïve or previously treated with ERT), and obstetric history. Obstetric history was not available for all women. We also examined clinical characteristics at study baseline and at last follow‐up prior to pregnancy (hemoglobin level, platelet count, spleen volume, and liver volume). Age at start of pregnancy and estimated time on eliglustat during pregnancy were estimated based on the reported date of last menstrual period. For two patients from the phase 2 trial, date of last menstrual period was not reported but was estimated by the investigator.

For female partners of men with GD1 who participated in phase 2 and phase 3 clinical trials and became pregnant during the trials, no data other than pregnancy outcome were available. For the one woman without GD1 who became pregnant in a phase 1 trial, only pregnancy outcome, information related to pregnancy‐associated adverse events, and obstetric history were available.

All reported pregnancies were followed until the outcome of the pregnancy was known. The infants were not followed as part of trial protocol, and thus potential long‐term health effects of exposure during pregnancy are unknown.

## 3 RESULTS

### 3.1 Pregnancies in female patients with GD1 in phase 2 and phase 3 trials

**FIGURE 1:**
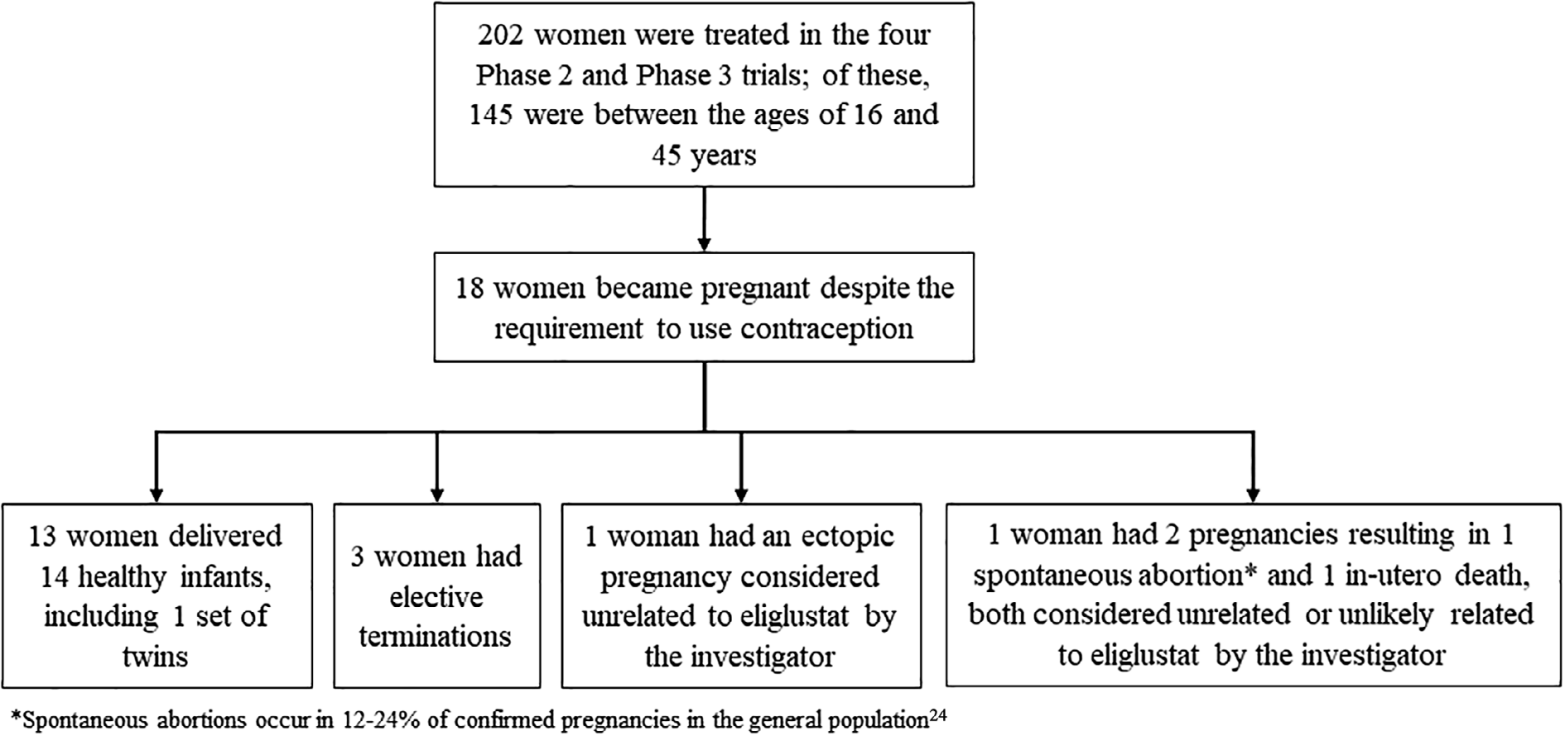
Patient disposition

Of 202 female patients with GD1 participating in the phase 2 and phase 3 trials, 145 (72%) were aged 16 to <45 years, and 18 (all within this age range) became pregnant despite the requirement to use contraception (Figure 1). Baseline characteristics of these 18 women are shown in Table 1. Two‐thirds of the women had a genotype with at least one p.Leu483Pro (L444P) allele, which is associated with more severe baseline disease manifestations.^1^ Their mean age at diagnosis of GD1 was 15.5 years, which is lower than the mean age of 20.5 years for the women in the combined phase 2 and phase 3 clinical trials population. Four women were treatment‐naïve prior to starting their trial, and the remaining women were previously treated with ERT prior to beginning eliglustat in their trial.

**TABLE 1:** Baseline characteristics of 18 women with GD1 who became pregnant in a phase 2 or phase 3 eliglustat clinical trial

Characteristic	N = 18
Age in years (mean ± SD)	
At diagnosis of Gaucher disease	15.5 ± 6.2
At first eliglustat exposure	24.5 ± 5.2
At beginning of pregnancy^a^	26.5 ± 4.8
Days on eliglustat treatment, median [25th, 75th percentile]	511.5 [257, 1250]
Days on eliglustat treatment during pregnancy^a^, median [25th, 75th percentile]	38.0 [30, 53]
CYP2D6 metabolizer genotype, n (%)	
Poor	1 (6%)
Intermediate	2 (11%)
Extensive	14 (78%)
Indeterminate	1 (6%)
Race, n (%)	
Caucasian	12 (67%)
Asian	4 (22%)
Other/unknown	2 (11%)
Ashkenazi Jewish descent, n (%)	1 (6%)
Hispanic or Latino ethnicity, n (%)	7 (39%)
Gaucher disease genotype, n (%)	
p.Asn409Ser/p.Asn409Ser (N370S/N370S)	1 (6%)
p.Asn409Ser/Other (N370S/other)	3 (17%)
p.Asn409Ser/p.Leu483Pro (N370S/L444P)	7 (39%)
p.Leu483Pro/p.Asp448His (L444P/D409H)	1 (6%)
p.Leu483Pro/other (L444P/other)	4 (22%)
Other/other	2 (11%)
Splenectomized, n (%)	4 (22%)

^a^ Age at beginning of pregnancy and days on eliglustat during pregnancy estimated based on the 19 pregnancies that occurred during the trials. Date of last menstrual period was not available for 2 women; age at pregnancy was estimated with available data.

In total, 19 pregnancies occurred in the 18 women. Pregnancy outcomes and clinical characteristics in these women are shown in Table 2. Thirteen women delivered 14 healthy infants, including one set of twins. Ten of these 13 women had a cesarean section (C‐section). Three women elected to terminate the pregnancy (reasons unknown); obstetric history is unknown for these three women. One woman had an ectopic pregnancy that was considered unrelated to eliglustat by the investigator; she had no history of previous pregnancies. One woman became pregnant twice. Her first pregnancy resulted in spontaneous abortion, and the second pregnancy resulted in in utero death; these were considered unrelated and remote/unlikely related to eliglustat, respectively, by the investigator. She had no history of previous pregnancies. In these 18 women, overall exposure to eliglustat during the trials ranged from 117 to 1537 days, and exposure to eliglustat during pregnancy ranged from 0 to 101 days (Tables 1 and 2). The woman included with 0 days of eliglustat exposure during pregnancy stopped eliglustat before she became pregnant but did not leave the trial until after pregnancy was confirmed. Her systemic exposure would have been minimal at the time of conception, given the short plasma elimination half‐life of eliglustat (this woman was a CYP2D6 extensive metabolizer; elimination half‐life is approximately 4 to 7 hours in nonpoor metabolizers^4^). The woman with the longest estimated exposure to eliglustat during pregnancy (time between last reported negative and positive pregnancy tests)had a negative pregnancy test at a scheduled site visit and was confirmed pregnant at the following scheduled site visit 3 months later.

**TABLE 2:** Pregnancy outcomes and clinical characteristics of 18 women with GD1 who became pregnant in a phase 2 or phase 3 eliglustat clinical trial

Age in years at start of pregnancy^a^ (range)	Gaucher genotype^b^	CYP2D6 metabolizer status	Days on eliglustat during the trial	Estimated days on eliglustat while pregnant^c^	Withdrew from trial?	Clinical status at last follow‐up before pregnancy^d^				Pregnancy outcome^e^
						Hemoglobin (g/dL)	Platelet count (×10^9^/L)	Spleen volume (MN)	Liver volume (MN)	
Treatment‐naïve patients										
21‐25	p.Leu483Pro/p.Asp448His	Extensive	184	30	Yes	10.7	56	18.31	2	Healthy infant delivered at 39 weeks via C‐section
	p.Asn409Ser/Other	Extensive	1044	53	Yes	11.05	134	3.73	1.07	Healthy infant delivered at 39 weeks
26‐30	p.Leu483Pro/Other	Extensive	94	28	No	9.8	57	59.7	2.11	Spontaneous abortion^f^
			385	101	Yes	8.9	44	48.77	2.07	In utero death at 37 weeks
	p.Asn409Ser /Other	Extensive	117	35	Yes	9.7	53	33.95	1.99	Healthy infant delivered via C‐section
Patients previously on ERT										
≤20	Other/Other	Intermediate	284	87	Yes	12.35	217.5	N/A*	1.33	Elective termination
	p.Asn409Ser/p.Leu483Pro	Extensive	344	41	Yes	13.8	255	1.06	0.85	Healthy infant delivered at 33 weeks via C‐section
21‐25	p.Leu483Pro/Other	Intermediate	1537	2	No^g^	11.1	310	N/A*	1.07	Healthy infant delivered
	p.Asn409Ser/p.Leu483Pro	Extensive	1453	0^h^	Yes	13.4	163	3.04	0.86	Healthy infant delivered at 39 weeks via C‐section
	p.Leu483Pro/Other	Extensive	257	59	Yes	12.4	149	4.41	1.05	Elective termination
	p.Asn409Ser/p.Leu483Pro	Poor	594	50	Yes	13.2	270	N/A*	1.25	Ectopic pregnancy
	p.Asn409Ser/p.Asn409Ser	Extensive	1536	39	Yes	13.1	171	2.35	1.16	Healthy infant delivered at 40 weeks via C‐section
	p.Asn409Ser/p.Leu483Pro	Extensive	1250	35	No^e^	11.9	169	1.64	0.98	Healthy infant delivered
26‐30	p.Asn409Ser/Other	Extensive	253	38	Yes	12.8	92	4.07	1.26	Healthy infant delivered at 38 weeks via C‐section
	p.Asn409Ser/p.Leu483Pro	Extensive	1092	32	Yes	13	172	1.63	0.96	Healthy infant delivered via C‐section
	Other/Other	Indeterminate	138	36	Yes	10.9	145	8.53	1.57	Elective termination
31‐35	p.Asn409Ser/p.Leu483Pro	Extensive	1333	43	Yes	12.5	122	4.9	1.24	Healthy infant delivered at 37 weeks via C‐section
36‐40	p.Leu483Pro/Other	Extensive	1164	10	Yes	14.8	196.5	2.27	0.74	Healthy twins delivered at 34 weeks via C‐section
	p.Asn409Ser/L444Pp.Leu483Pro	Extensive	429	96	Yes	12.9	235	N/A*	0.9	Healthy infant delivered at 38 weeks via C‐section

Abbreviations: ERT, enzyme replacement therapy; MN, multiples of normal; N/A*, patient is splenectomized.

^a^ Updated genotype nomenclature: pLeu483Pro = L444P; p.Asn409Ser = N370S; p.Asp448His = D409H.

^b^ Age at start of pregnancy and estimated days on eliglustat while pregnant determined by the reported or estimated date of last menstrual period. Actual ages are not provided to protect patient privacy.

^c^ Estimated by the difference of reported or estimated date of last menstrual period and date when drug was discontinued.

^d^ Not all participants had clinical assessment measured close to the time pregnancy was detected. Some values were measured on the same visit that pregnancy was identified.

^e^ Gestational age was not available for all deliveries.

^f^ Spontaneous abortions occur in 12% to 24% of confirmed pregnancies in the general population.^24^

^g^ Patient became pregnant at the end of the clinical trial and was not considered to be a study withdrawal.

^h^ Patient discontinued eliglustat before she became pregnant but did not leave the trial until after pregnancy was confirmed.

The woman who became pregnant twice subsequently died of surgical complications 7 months after leaving the trial. She was treatment‐naïve prior to the trial and had severe GD1, as denoted by her anemia, moderate to severe thrombocytopenia, massive splenomegaly, and moderate hepatomegaly. Her first pregnancy was not detected until after spontaneous abortion, which was considered unrelated to eliglustat by the investigator. Estimated exposure to eliglustat during pregnancy was less than 4 weeks. After the spontaneous abortion, eliglustat treatment was interrupted for 3 days and then restarted. The patient's case was discussed with the principal investigator and independent data monitoring committee; the patient was no longer pregnant since spontaneous abortion had occurred, and the patient needed treatment for her severe GD1, so she was not withdrawn from the trial. Her second pregnancy started approximately 9 months after the first pregnancy. She discontinued eliglustat after pregnancy was confirmed (a sonogram estimated the gestational age of the fetus to be 18 ± 2 weeks) and withdrew from the trial. The patient lived in a country for which use of imiglucerase (the only ERT available at the time) during pregnancy was, at the time, off‐label. Thus, after leaving the trial, she was untreated for the remainder of her second pregnancy. Mild metrorrhagia and mild hypertension were reported at 33 weeks' gestation, and while admitted in the hospital the patient was started on methyldopa treatment 500 mg three times a day. Intrauterine death was revealed by ultrasound 1 month later at 37 weeks' gestation, which was considered unlikely to be related to eliglustat by the investigator. During C‐section the next day, the patient experienced moderate bronchospasm, mild cutaneous rash, and moderate edema of the eyelids. The patient recovered without sequelae from all events. Fetal autopsy was requested, but the patient refused consent. The patient was started on imiglucerase 1 month after the fetal death. One month later, she died of surgical complications: hypovolemic shock due to splenic rupture after a laparoscopic cholecystectomy for gallstones, which are common in patients with Gaucher disease.^23^ Her death, which occurred 7 months after eliglustat was discontinued, was considered unrelated to eliglustat by the investigator.

### 3.2 Pregnancies in female partners of male patients with GD1 in phase 2 and phase 3 trials

A total of 18 pregnancies were reported in the partners of 16 male patients with GD1 during the time these patients were receiving eliglustat in a phase 2 or phase 3 trial. All 18 pregnancies resulted in delivery of healthy infants. In addition, one female partner had two earlier pregnancies while the male patient was receiving placebo during the ENGAGE phase 3 trial. The first pregnancy resulted in a spontaneous abortion, and the second pregnancy resulted in delivery of healthy twins. Overall exposure to eliglustat during the trials for the 16 men was a median of 3.6 years.

### 3.3 Pregnancy in a woman without GD1 in a phase 1 trial

Similar to the phase 2 and phase 3 trials, all female subjects in phase 1 trials were required to use contraception. One pregnancy occurred in a 20‐year‐old female subject without GD1 during a phase 1 drug‐drug interaction study of eliglustat and acid‐reducing agents. The subject received one 84‐mg dose of eliglustat concomitantly with Maalox (1600‐mg aluminum hydroxide, 1600‐mg magnesium hydroxide, 160‐mg simethicone), followed 8 days later by one 84‐mg dose of eliglustat. This subject would not have reached steady state of eliglustat given that the two doses of eliglustat were separated by 8 days. Pregnancy tests conducted prior to each dose were negative. The woman had a positive pregnancy test 5 days after the second dose of eliglustat and was discontinued from the study. Fourteen days after the positive pregnancy test, she had a spontaneous abortion, which was considered possibly related to eliglustat and unrelated to Maalox by the investigator. The subject had a history of one previous spontaneous abortion and one full‐term live birth.

## 4 DISCUSSION

In total, 38 pregnancies were reported in eliglustat clinical trials, with 19 pregnancies reported in 18 of the 202 female patients with GD1 receiving eliglustat in phase 2 or phase 3 clinical trials, 18 pregnancies in the female partners of 16 male patients with GD1 taking eliglustat in phase 2 or phase 3 trials, and one pregnancy in a healthy female subject who participated in a phase 1 eliglustat trial. Most pregnancies that were continued in the group of 18 female patients with GD1 resulted in delivery of healthy infants (13/16). Similarly, all 18 pregnancies in female partners of the 16 male patients with GD1 in eliglustat trials resulted in delivery of healthy infants. The only pregnancy reported in a healthy volunteer female subject participating in a phase 1 drug‐drug interaction study resulted in a spontaneous abortion.

The 18 female patients with GD1 who became pregnant had, on average, more severe Gaucher disease than all female patients in the full combined clinical trial population (n = 202), as evidenced by the high proportion of women with a p.Leu483Pro (L444P) allele and a lower mean age at diagnosis. Twelve (67%) of the 18 female patients with GD1 who became pregnant during the phase 2 and phase 3 trials had at least one p.Leu483Pro (L444P) allele, while 81/202 (40%) female patients in the combined clinical trials population had at least one p.Leu483Pro (L444P) allele. The mean age at GD1 diagnosis in the 18 women was 15.5 years, which is younger than the mean age at diagnosis of 20.5 years for all female patients in the combined clinical trials population.

Spontaneous abortion has been reported to occur in 12% to 24% of confirmed pregnancies in the general population^24^ and was reported in 3.6% of untreated Gaucher patients and 6.9% of ERT‐treated patients in a recent report from the Gaucher Outcome Survey.^9^ In the phase 1, 2, and 3 trials, spontaneous abortion occurred in 1 (5%) out of 19 pregnancies in women with GD1 who received eliglustat and in 1 female participant without GD1 who became pregnant during a phase 1 trial. A high proportion of the women with GD1 (10/13) who had a live birth delivered by C‐section. The patient who had two pregnancies and subsequently died of complications following cholecystectomy highlights the risk of untreated GD1 and treatment interruptions, especially in the context of pregnancy and severe GD1.

This analysis is descriptive and reflects only short‐term exposure to eliglustat during pregnancy, because women who became pregnant during their trial had to stop eliglustat treatment. A second major limitation of this analysis is that infants were not followed as part of trial protocol, so there is no information on the long‐term outcomes of the infants. In addition, there is limited information on the pregnancies that occurred in the female partners of male patients with GD1 treated with eliglustat in the trials, including lack of information on the women's age and clinical status and infants' gestational age at birth. Thus, no conclusions can be drawn from this data with respect to the use of eliglustat during pregnancy, and no changes can be made or inferred with regard to the recommendations in the drug label that eliglustat should be avoided during pregnancy. Comparatively, there are much more data on the use of ERT during pregnancy, which can be used throughout pregnancy.^4^ Close monitoring of the pregnancy and clinical manifestations of Gaucher disease is necessary as patients may experience a period of increased disease activity during pregnancy and the puerperium, including risk of skeletal manifestations, exacerbation of cytopenia, hemorrhage, and increased need for transfusion. Close follow‐up and data collection are also needed when pregnant women discontinue eliglustat in order to understand the potential impact of drug exposure.

With regard to male patients with GD1, in this analysis, all 18 partner pregnancies resulted in delivery of healthy infants. Although these encouraging data suggest that eliglustat exposure does not affect male fertility, no firm conclusion can be drawn as the trials did not specifically address the impact of eliglustat on male fertility, and again, long‐term outcome of the infants is unknown. Of note, miglustat, a second‐line oral substrate‐reduction therapy for adults with GD1, is also not recommended during pregnancy and was found to reduce male fertility in animals,^25^ although a subsequent study in humans did not corroborate this finding.^26^ However, miglustat and eliglustat have different specificity, tolerability, and safety profiles^21^, ^25^ and are structurally different; miglustat is an iminosugar analogue whereas eliglustat is a ceramide analogue. Thus, pregnancy and fertility outcomes associated with miglustat cannot be assumed to apply also to eliglustat.

The purpose of this manuscript was to share available information on pregnancy outcomes of the small subset of women with GD who became pregnant while taking eliglustat to aid clinicians and patients in decision‐making. Guidelines for clinicians and patients with GD that address use of eliglustat in women of childbearing potential are needed.

## AUTHOR CONTRIBUTIONS

Design of the study was done by Meredith C. Foster and M. Judith Peterschmitt. Interpretation of data was done by all authors. Meredith C. Foster performed data analysis. Grace Lewis drafted the manuscript, and all authors critically revised the manuscript. All authors approved the final manuscript.

## DISCLOSURE OF INTERESTS

Elena Lukina was a principal investigator in eliglustat clinical trials and has received honoraria, consulting fees, and travel reimbursement from Sanofi Genzyme and Shire; Manisha Balwani and Nora Watman were principal investigators in eliglustat clinical trials and receive honoraria and travel reimbursement from Sanofi Genzyme; Nadia Belmatoug has received consulting fees from Sanofi Genzyme and Shire; Assistance Publique—Hôpitaux de Paris Nord has received research funding from Shire/Takeda and Sanofi Genzyme; Derralynn Hughes has received research funding and consulting fees from Sanofi Genzyme; Sebastiaan J. M. Gaemers, Meredith C. Foster, and M. Judith Peterschmitt are employees of Sanofi Genzyme; Grace Lewis is a PharmD fellow at Sanofi Genzyme.

## ETHICS STATEMENT

The protocols for all studies were approved by the institutional review boards or independent ethics committees of participating institutions.

## Data Availability

Requests to analyze data sets used in the current study can be submitted to http://ClinicalStudyDataRequest.com
